# Finding the Integrated Care Evidence Base in PubMed and Beyond: A Bibliometric Study of the Challenges

**DOI:** 10.5334/ijic.3975

**Published:** 2018-08-17

**Authors:** Suzanne Lewis, Raechel A. Damarell, Jennifer J. Tieman, Camilla Trenerry

**Affiliations:** 1 Central Coast Local Health District, New South Wales, AU; 2 Flinders University, South Australia, AU

**Keywords:** Bibliometrics, PubMed, integrated care research, search filters, literature searching

## Abstract

**Introduction:**

Integrated care research evidence should be optimally visible and accessible to stakeholders. This study examines the contribution of specific databases to the discovery of integrated care evidence, and tests the usefulness of Medical Subject Heading (MeSH) indexing of this literature within PubMed.

**Methods:**

We used bibliometric methods to analyse the integrated care literature indexed within six databases between 2007 and 2016. An international expert advisory group assessed the relevance of citations randomly retrieved from PubMed using MeSH term ‘Delivery of Health Care, Integrated’.

**Results:**

Integrated care evidence is diffuse, spread across many journals. Between 2007 and 2016, integrated care citations grew substantially, with the rate of increase highest in Embase. PubMed contributes the largest proportion of unique citations (citations not included in any of the other databases analysed), followed by Embase, PsycINFO and CINAHL. On average, expert reviewers rated 42.5% of citations retrieved by MeSH term ‘Delivery of Health Care, Integrated’ as relevant to integrated care. When these citations were dual reviewed, inter-rater agreement was low.

**Conclusion:**

MeSH terms alone are insufficient to retrieve integrated care content from PubMed. Embase and CINAHL contain unique content not found in PubMed that should not be overlooked. A validated search filter is proposed to simplify the process of finding integrated care research for clinicians, managers and decision-makers.

## Introduction

Integrated care is an overarching approach to delivering care within and beyond the health sector, which aims to achieve high quality services, improved patient experience and efficiency, by placing the patient, their family and community at the centre of care. It has become increasingly important in health care policy and practice as a means to address growing health demands, consumer expectations, and the reality of managing chronic disease, ageing and multimorbidity. However, despite its global importance, integrated care still lacks a universally agreed upon definition and the terminology used to describe it remains varied and highly contextual [1,2,3]. If integrated care is considered essential for optimising patient-centred healthcare experiences and outcomes, as well as containing the burgeoning costs associated with care, its evidence base needs to be optimally accessible and visible to stakeholders.

The purpose of this paper is to present the findings of two related research activities. The first was a bibliometric analysis of the published integrated care literature, undertaken in order to better understand the scholarly publishing landscape in relation to integrated care. The second research activity sought to gauge the usefulness of the Medical Subject Heading (MeSH) ‘Delivery of Health Care, Integrated’ in locating relevant integrated care literature indexed in the PubMed database. In order to address the issues identified in this research, a validated search filter is proposed to facilitate access to the published literature on integrated care.

## Theory and Methods

### Definitional issues

As early as 2002, Kodner and Spreeuwenberg noted that “lack of conceptual clarity stands as a major barrier to promoting integrated care in both theory and practice” [4, p. 1]. They proposed two ways of approaching integration: a top-down, hierarchical approach derived from systems theory and a bottom-up, patient-centred approach from which their following definition is derived.

“Integration is a coherent set of methods and models on the funding, administrative, organisational, service delivery and clinical levels designed to create connectivity, alignment and collaboration within and between the cure and care sectors. The goal of these methods and models is to enhance quality of care and quality of life, consumer satisfaction and system efficiency for patients with complex, long term problems cutting across multiple services, providers and settings. The result of such multi-pronged efforts to promote integration … is called ‘integrated care’” [4, p. 3].

Kodner revisited the concept of integrated care in 2009 and noted that definitions had multiplied rather than consolidated since 2002 [5]. Despite increasing imperatives to apply integration to public health systems, initiatives were still hampered by the absence of a common definition of, and confusing terminology relating to, integrated care. Furthermore, a number of terms commonly equate with integrated care. These include: managed care, continuity of care, case/care management, transmural care, patient-centred care, seamless care, collaborative care, transitional care and integrated delivery systems [5,6].

Numerous attempts have been made to develop taxonomies and conceptual frameworks to clarify the domains of integrated care [7,8,9,10,11]. These taxonomies elucidate the multiple layers, players, and facets that should be considered part of integrated care strategies, while simultaneously highlighting the complexities involved [12]. Despite this complexity, health professionals, policy makers and consumers need to be able to access the empirical evidence supporting integrated care’s efficacy and value if its potential contribution to improved care is to be realised.

### Literature searching issues

Searching for integrated care research literature is complex. As described above, there is a substantial vocabulary used to describe integrated care programs, settings, initiatives and evaluations in the published literature. For example, one literature review identified over 70 terms and phrases related to integrated care, which then yielded 175 definitions and concepts [13].

Integrated care is also a multidisciplinary activity that takes place across multiple levels of organisations and systems [14] and addresses multiple audiences. The net effect is to spread, rather than concentrate, the literature across a wide range of academic journals, making it diffuse and complicating the process of finding it. A range of subject areas lying outside of health may also inform integrated care, for example social welfare, education, economics, and information technology [15]. This requires searchers to be aware of specialist subject databases beyond health databases. Bibliographic databases are sophisticated commercial products requiring a minimum level of technical expertise on the part of the searcher. Discipline knowledge is often not enough to guarantee success in the efficient retrieval of relevant literature [16]. Effective searching across a range of databases is dependent on a searcher’s level of knowledge of the various interfaces, syntaxes, indexing practices, and search algorithms involved. Several systematic review authors have documented the specific difficulties in searching for integrated care literature [15,17]. They note that while a database search strategy developed for a clinical question in a biomedical database can be “specific, precise and unambiguous” [15, p. 81], this is not always the case in databases indexing the literature in related fields. Non-specific search terms and varying definitions demand broad, inclusive search strategies which retrieve a high proportion of irrelevant citations.

Compounding the difficulties involved in identifying integrated care literature is the existence of a considerable body of integrated care knowledge lying outside academic journals within the grey literature. Grey literature is defined as “…information produced on all levels of government, academia, business and industry in electronic and print formats not controlled by commercial publishing” [18].

Grey literature includes resources such as conference papers, reports, newsletters, emails, blogs, websites, and government documents, which may lack a stable URL, secure archiving, or may be part of the deep web, inaccessible even to search engines such as Google. More recently, improvements in search engines and open access publishing mean that grey literature is much more visible, but the searcher risks being overwhelmed by the number of sources and the variable quality and relevance of the results.

### PubMed issues

PubMed (www.pubmed.gov), a free service of the US National Library of Medicine (NLM), is arguably the foremost international database indexing the biomedical literature and is therefore important in the consideration of retrieval of integrated care literature. Despite a simple search interface, certain features of PubMed’s query translation algorithm may result in end users obtaining less than optimal search results [16,19]. As a North American funded resource its coverage of the broader international research [20], especially that produced in low to middle-income countries, can be limited.

This North American bias is also reflected in PubMed’s controlled thesaurus of MeSH used to describe the majority of articles included in the database. The MeSH term most closely describing integrated care, ‘Delivery of Health Care, Integrated’, has a definition aligned with the concept of managed care (https://www.ncbi.nlm.nih.gov/mesh/68019033). Managed care, while highly relevant to the North American healthcare system, may be less appropriate for other healthcare delivery locations. The MeSH term list is updated annually and one of the new terms added in 2017 was ‘Intersectoral Collaboration’, defined as “Cooperative actions and ventures among health and health-related groups and organizations intended to improve health outcomes” [21]. This term may hold promise for the future as a more accurate descriptor of integrated care as it is conceptualised and practised in countries such as the United Kingdom, Australia, Canada, New Zealand and elsewhere.

It is also worth noting that three key integrated care journals, the *International Journal of Integrated Care* (ISSN 1568-4156), the *Journal of Integrated Care* (ISSN 1476-9018) and the *International Journal of Care Coordination*, (ISSN 2053-4345) formerly the *Journal of Integrated Care Pathways*, are not fully searchable within the PubMed database. Citations for the *International Journal of Integrated Care* lack MeSH term indexing so cannot be retrieved using ‘Delivery of Health Care, Integrated’. They are discoverable via keyword searching of title and abstract fields but as of January 2018, a relatively high proportion (147/568; 26%) also lack abstracts, making them retrievable only by terms in the article title. These articles are effectively lost if their titles are not sufficiently descriptive. It is to be hoped that the *International Journal of Integrated Care* will be accepted by NLM for PubMed Medline indexing in the future in order to maximise the discoverability of its highly relevant content. Citations from the *Journal of Integrated Care* and the *International Journal of Care Coordination* are not included in PubMed except for a very small number where author manuscripts have been deposited in PubMed Central.

### Bibliometric analysis

Bibliometrics is the quantitative activity of tracking the body of literature produced within a specific field, chiefly its growth and dissemination patterns, and the degree to which it is referred to across the scholarly record. Bibliometric analysis can complement expert input in increasing understanding of the published literature relevant to a field of research or practice, particularly complex, fluid and diffuse fields [22,23]. A previous bibliometric analysis of the integrated care literature by Sun and colleagues investigated the growth of research in the field and identified the key journals and research domains of interest [24]. That study focused only on PubMed. It described a steep increase in the number of articles on integrated care published since 1993 in a broad range of subject specific journals and described by a large number of keywords (5875), 50% of which had a single occurrence [24].

### Search filters

A search filter is an objectively derived search string with known retrieval effectiveness, which enables brokered access to the indexed content within a particular bibliographic database. Search filters are already well-established tools for finding research evidence by study methodology [25,26,27]. They are also increasingly useful for ensuring reliable, rapid access to literature on conceptually complex subject areas [28,29,30,31]. To our knowledge, no subject search filter has been developed for integrated care prior to our study.

## Methods

A bibliometric analysis of the integrated care literature was undertaken, involving analyses of PubMed content, as well as the content of a range of databases beyond PubMed. All analyses were restricted to the year range 2007–2016 to ensure near-completeness of coverage across the span of 10 years. No language restrictions were applied.

### PubMed methods

Within PubMed we specifically investigated:

1. the prevalent MeSH terms used to describe articles containing integrated care terminology;
2. the prevalent PubMed journal titles conveying integrated care content; and
3. the prevalent countries of publication.

The open access text mining tool PubMed PubReminer (http://hgserver2.amc.nl/cgi-bin/miner/miner2.cgi) was used for the PubMed analysis. This is one of several front-ends available to analyse PubMed search results. It produces frequency tables ranking the number of occurrences of each year of publication, journal, author, keyword, MeSH term and country of publication [32].

The following search strategy was used in PubReminer:

(Integrated care[tiab] OR integrated service*[tiab] OR integrated health*[tiab] OR integrating health*[tiab] OR “integration of care”[tiab] OR “integration of services”[tiab] OR integrating care[tiab] OR care integration[tiab]) AND 2007/01/01[PDat]:2016/12/31[PDat].

Search terms were restricted to the title and abstract fields only. There were several reasons for this:

- to avoid the search picking up MeSH terms that may not be relevant to the topic;
- to avoid adding ‘weight’ to journals indexed with MeSH terms; and
- to create a search that can be equitably translated to subsequent databases that may not include indexing terms, or may include indexing terms different to those used by PubMed.

Alternative terms to the most obvious ‘integrated care’ were included in the strategy to ensure retrieval of as much of the integrated care literature as possible. These terms were gleaned from search strategies of existing published systematic reviews on the topic of integrated care. This is a point of departure from Sun and colleagues’ bibliometric study which used a single MeSH term and the single keyword ‘integrated care’ in its strategy [24].

The search was restricted to literature published between 2007 and 2016, including indexed and non-indexed PubMed content. This is a further point of difference with the Sun study which applied no date restrictions.

Results were saved as a text file and edited in Excel. The following categories were retained in the spreadsheet for analysis: publication year, journal title, MeSH term, and country. This search was run on 5 January 2018.

### Databases beyond PubMed

Using databases Embase (Ovid), CINAHL (EBSCOhost), PsycINFO (Ovid), Cochrane Central Register of Controlled Trials, and Econlit (Ovid), we sought to answer these questions:

1. What is the relative proportion of unique citations provided by databases other than PubMed and are these databases useful contributors?
2. What is the rate of growth in integrated care content across databases?
3. Which are the prevalent journal titles publishing integrated care literature when all databases are taken into account?

To identify the relative contribution of integrated care literature across other key databases, a PubMed search strategy was accurately translated for five additional databases. Table 1 shows the databases and search strategies used.

**Table 1:** Search strategies used to retrieve integrated care literature.

Database	PubMed	CINAHL (EBSCOhost)	Embase, PsycINFO, and Econlit (Ovid)	CENTRAL (Cochrane Central Register of Controlled Trials)

Search strategy	(Integrated care[tiab] OR integrated service*[tiab] OR integrated health*[tiab] OR integrating health*[tiab] OR “integration of care”[tiab] OR “integration of services”[tiab] OR integrating care[tiab] OR care integration[tiab]) AND 2007/01/01[PDat]:2016/12/31[PDat]	TI ((“Integrated care” OR “integrated service*” OR “integrated health*” OR “integrating health*” OR “integration of care” OR “integration of services” OR “integrating care” OR “care integration”)) OR AB ((“Integrated care” OR “integrated service*” OR “integrated health*” OR “integrating health*” OR “integration of care” OR “integration of services” OR “integrating care” OR “care integration”)) Limited to 2007–2016	(Integrated care OR integrated service* OR integrated health* OR integrating health* OR integration of care OR integration of services OR integrating care OR care integration).ti,ab. limit 1 to yr = “2007–2016”	(“Integrated care” OR “integrated service*” OR “integrated health*” OR “integrating health*” OR “integration of care” OR “integration of services” OR “integrating care” OR “care integration”) Search limited to Record Title or Abstract and 2007–2016

Notes on search syntax:
[tiab] = PubMed title and abstract field search. TI = CINAHL title field search. AB = CINAHL abstract field search. .ti,ab = Ovid database syntax for title and abstract field search. The truncation mark * at the end of a word stem allows for retrieval on all variants of that word stem. For example, health* retrieves *health* OR *healthcare*.

Each database’s results were exported into an EndNote Library set up for that database. These sets were used to ascertain absolute growth in citations by database for the following year ranges: 2007–2008; 2009–2010; 2011–2012; 2013–2014; 2015–2016.

Libraries were then set up to combine citations retrieved by the PubMed search with citations retrieved from each of the remaining five databases. This produced five libraries:

1. Embase vs PubMed;
2. CINAHL vs PubMed;
3. PsycINFO vs PubMed;
4. Central vs PubMed; and
5. Econlit vs PubMed.

Within each of these libraries, duplicate citation pairs, (i.e. those retrieved by both PubMed *and* the additional database), were identified and removed to leave only unique citations. The availability of a ‘Name of Database’ field in the EndNote record made it possible to then calculate the relative numbers of unique citations contributed by each database. The comparative prevalence of *unique* integrated care content in each database was then graphed across the set year ranges using PubMed as the absolute benchmark.

To calculate the total number of unique citations retrieved by all searches, irrespective of originating database, all citations retrieved by each database were merged into one library (‘Total Unique Set’) and duplicates removed. This time, however, only subsequent copies of a duplicated citation were removed (not duplicate pairs etc.). This created a complete set of unique citations which was then used to identify the prevalent journals titles and the diffusion of citations across all journal titles for 2007–2016.

### Expert-rated usefulness of MeSH term ‘Delivery of Health Care, Integrated’

A further exercise was undertaken to assess the usefulness of the MeSH term with the most face validity: ‘Delivery of Health Care, Integrated’. For this, a search was executed in PubMed using only this MeSH term. Sets of 100 citations were randomly generated from the results for the publication years 2010, 2013 and 2016. Sets were distributed to 12 international experts on integrated care for their review. These individuals had been identified by Dr Nick Goodwin, Chief Executive Officer of the International Foundation for Integrated Care, and by the authors, and invited to join the project in an advisory role. In response to the question “Does this article describe integrated care?’ the experts were instructed to select yes, no or unsure for each citation.

## Results

### PubMed’s contribution

The search of PubMed using PubReminer retrieved 5005 citations and showed a growth in articles from 279 in 2007 to 876 in 2016.

Of the top 10 highest frequency MeSH terms:

- six were generic MeSH subheadings rather than MeSH terms: organization & administration; epidemiology; economics; statistics & numerical data; therapy; methods;
- three were generic MeSH terms that serve as PubMed filters: humans, female, male;
- only one was a serviceable MeSH term specific to the topic of interest: ‘Delivery of Health Care, Integrated’.

The next meaningful MeSH term was ‘Primary Health Care’ ranked at number 24 in the count.

**Figure 1:**
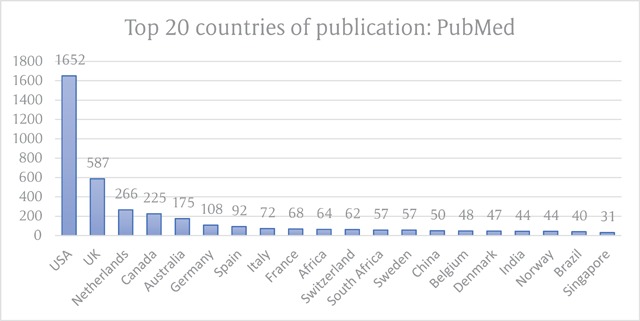
Top 20 ranked countries of publication for integrated care literature in PubMed.

The United States dominated the country of publication (n = 1652; 33.0%), followed by the United Kingdom (587; 11.7%), and the Netherlands (266; 5.3%). The top 20 countries of publication are shown as Figure 1.

The top 20 journal titles identified by the PubMed search are shown in Table 2. The scope of many of these journals is broad, reflecting the multifaceted and multidisciplinary nature of integrated care. Three of these titles, including the most prevalent one, are not indexed with MeSH terms in PubMed. This means their content is not discoverable using a MeSH search alone.

**Table 2:** Top 20 journal titles publishing articles on integrated care in PubMed.

Journal	Number of citations	MeSH-indexed in PubMed? (Y/N)

International Journal of Integrated Care	254	N
BMC Health Services Research	98	Y
Psychiatric services (Washington, DC)	56	Y
Journal of General Internal Medicine	51	Y
The American Journal of Managed Care	46	Y
^±^Studies in Health Technology and Informatics	43	Y
BMJ (Clinical Research Ed.)	38	Y
PLoS One	36	Y
Medical Care	35	Y
BMC Public Health	34	Y
London Journal of Primary Care	32	N
Journal of the American Geriatrics Society	30	Y
Families, Systems & Health: The Journal of Collaborative Family Healthcare	29	Y
Health Affairs (Project Hope)	29	Y
Health Policy (Amsterdam, Netherlands)	28	Y
The Health Service Journal	28	N
BMJ Open	27	Y
The Permanente Journal	26	Y
Healthcare Quarterly (Toronto, Ont.)	21	Y
Psychiatrische Praxis	21	Y

^±^ Only articles on health technology assessments (HTAs) are currently MeSH-indexed.

### Contribution of databases beyond PubMed

The contribution of integrated care literature has increased substantially over the period 2007 to 2016 in PubMed and Embase, with the rate of increase highest in Embase. Modest growth is evident in databases CINAHL and PsycINFO, and very slight growth in Central and Econlit.

**Figure 2:**
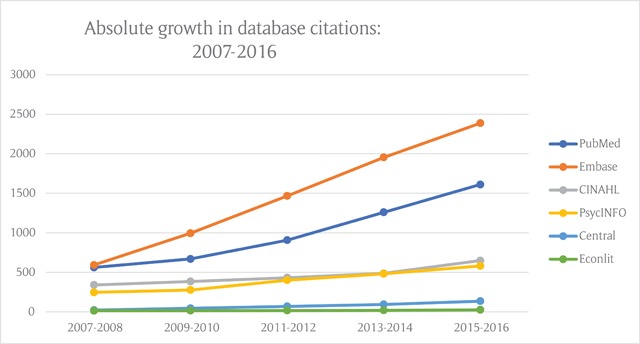
Absolute growth rate in citations for all databases.

The absolute rate of growth in citations of potential relevance to integrated care is shown in Figure 2.

When citations retrieved from Embase, CINAHL, PsycINFO, Central, and EconLit are combined and deduplicated against the PubMed citations, it is clear that Embase contributes a significant number of unique citations if conference abstracts are included (75%). With conference abstracts excluded, Embase contributes a more modest 14.1%. PsycINFO also reduces its contribution if books, book chapters, and theses are discounted. Not surprisingly, the Cochrane Central Register of Controlled Trials contributes only 2.2% of the unique citations and Econlit even less at 0.9%. Table 3 shows the relative unique contributions of each database when deduplicated against PubMed.

**Table 3:** Contribution of unique citations by databases beyond a PubMed search.

Database	Total number of citations when combined with PubMed (duplicates included)	Total after duplicate pairs removed	Number of unique citations contributed by non-PubMed database		Number of unique journal citations* contributed by non-PubMed database	

			n	%	n	%

Embase	12,398	3637	2728	75.0	513	14.1
CINAHL	7448	4225	773	18.3	744	17.6
PsycINFO	6991	4809	897	18.7	636	13.2
Central	5420	4847	108	2.2	105	2.2
Econlit	5097	5038	62	1.2	45	0.9

* Excludes conference abstracts, proceedings, theses, books, book chapters, and reports.

**Figure 3:**
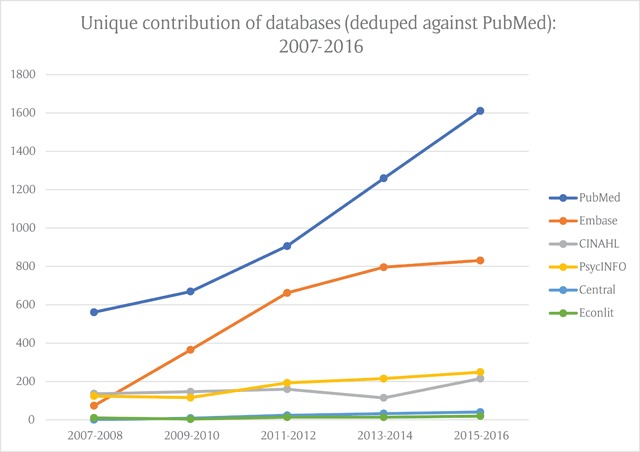
Rate of growth in unique citations by databases measured against a PubMed benchmark.

If PubMed is given precedence in any search for integrated care literature, and all subsequent database results are deduplicated against the PubMed results, the relative rate of growth in unique citations for Embase is less steep but still significant. (Figure 3). Once again, similar and modest contributions are made by CINAHL and PsycINFO, while Central and Econlit offer negligible additional citations to the total.

A total of 17,334 citations were combined in the ‘Total Unique Set’ EndNote Library. Once non-journal items were removed and the remaining citations deduplicated against each other, this reduced to 8844 citations. This constituted 2221 unique journal titles of which approximately half (49.6%) contributed only a single citation to the count. The mean number of citations per journal title was four.

Journal title frequency analysis of this combined set revealed the top 20 journals retrieved by the searches across the 6 databases (Table 4).

**Table 4:** Top 20 journal titles across all database searches.

Journal	Number of citations retrieved	Journal available in PubMed? (Y/N)

International Journal of Integrated Care	254	Y
Journal of General Internal Medicine	140	Y
Journal of Integrated Care (Brighton, England)	107	N^±^
BMC Health Services Research	99	Y
Journal of Clinical Oncology: Official Journal of the American Society of Clinical Oncology	91	Y*
Value in Health: The Journal of the International Society for Pharmacoeconomics and Outcomes Research	84	Y
Psychiatric services (Washington, DC)	71	Y
Journal of the American Geriatrics Society	59	Y
Pharmacoepidemiology and Drug Safety	58	Y
Mental Health Weekly	51	N
Annals of the Academy of Medicine, Singapore	48	Y*
BMJ (Clinical Research Ed.)	48	Y
Palliative Medicine	47	Y
The American Journal of Managed Care	46	Y
The European Respiratory Journal	46	Y*
Hepatology (Baltimore, Md.)	45	Y
Studies in Health Technology and Informatics	45	Y
American Journal of Respiratory and Critical Care Medicine	43	Y*
Circulation	42	Y*
Gastroenterology	39	Y
**Total number of citations**	**1463**	

^±^ An exception is author manuscripts deposited in PubMed Central (PMC) under compliance with mandated public access policies. * Supplements containing conference abstracts not included in PubMed.

Together these 20 journals contain 16.5% (1463/8844) of all retrieved content—rather a low concentration but not surprising considering the scattering of content across a large number of journals.

There are a number of significant differences between this list derived from all database retrievals and that produced from a PubMed search. Firstly, both lists only contain eight titles in common while a large number of specialist clinical journals appear in the multi-database search results and not the PubMed list (*Journal of Clinical Oncology, Palliative Medicine, European Respiratory Journal, Hepatology, American Journal of Respiratory and Critical Care Medicine, Circulation*, and *Gastroenterology*). These clinical journals are predominately citations for meeting abstracts indexed in the Embase database but not PubMed. Their inclusion in this list has the net effect of pushing out key health services research journals that appear in the PubMed list, including: *Families, Systems & Health; Health Affairs; Health Policy, Health Service Journal*, and *Healthcare Quarterly*.

*Pharmacoepidemiology and Drug Safety* appears in the multi-database list as its conference abstracts are well-indexed in Embase and a high proportion of its studies draws on patient population groups associated with integrated healthcare delivery systems such as Kaiser Permanente. This highlights a particular difficulty in searching for integrated care literature. Even the most specific search term, ‘integrated care’, can retrieve clinical studies conducted in this type of healthcare setting.

*Mental Health Weekly* appears in the multi-database search list as it has high issue publication frequency and comprises a large number of smaller news items featuring integrated care within a mental health context.

While these inclusions could be considered unhelpful to the searcher not interested in pursuing conference abstracts or clinical studies, the multi-database search list does contribute three additional titles with a comparatively high proportion of clearly relevant integrated care content. These are: *Journal of Integrated Care* (contributed by CINAHL), *Value in Health* (conference abstracts in Embase), and *Annals of the Academy of Medicine, Singapore* (conference abstracts in Embase). Thus, extending a literature search beyond PubMed to Embase and CINAHL will yield additional relevant content.

The *International Journal of Care Coordination* (formerly *International Journal of Care Pathways*) does not appear in the top 20 titles, despite its content being indexed in CINAHL and despite it being identified by members of our Expert Advisory Group as a key integrated care journal. This journal has undergone two changes of title during the period to which our analysis is restricted (2007–2016). If the title changes were to be absorbed into its current title it would rank at number 10.

### Expert-rated usefulness of MeSH term ‘Delivery of Health Care, Integrated’

Of the 12 experts invited to participate in this exercise, eight responded. Pairs of experts independently screened the 2010 and 2013 sets of citations retrieved by the MeSH term ‘Delivery of Health Care, Integrated’, while a group of four independently screened the 2016 set. Results are presented in Table 5 below.

**Table 5:** Results from expert screening of citations retrieved by MeSH term ‘Delivery of Health Care, Integrated’.

	Yes (Relevant)	No (Irrelevant)	Unsure	Missing response	Total

2010 Reviewer A	43	35	21	1	100
2010 Reviewer B	19	70	10	1	100
2013 Reviewer A	69	24	7	0	100
2013 Reviewer B	28	42	30	0	100
2016 Reviewer A	33	40	26	1	100
2016 Reviewer B	38	48	12	2	100
2016 Reviewer C	23	73	3	1	100
2016 Reviewer D	87	11	1	1	100

Results varied considerably with a maximum of 87 citations out of 100 being rated as describing integrated care and a minimum of 19 (average = 42.5, median = 35.5). When comparing the responses of two reviewers for any one particular year, the maximum number of citations rated by two reviewers as relevant to IC was 38 (Reviewer B and D, in 2016) and the minimum was 16 (2010). Inter-rater reliability calculations (Cohen’s Kappa = 0.271 for the 2010 set and 0.245 for the 2013 set; Fleiss’ Kappa = 0.161 for the 2016 set), demonstrate only slight-fair agreement according to a commonly used guide for interpreting kappa scores [33].

## Discussion

Our results confirm the findings of a previous bibliometric analysis [24] and support the experiences of reviewers of the integrated care literature [15,17]. The literature is indexed across a range of bibliographic databases, and the volume of published research in the field is increasing steadily. A substantial amount of the integrated care literature in PubMed is not discoverable in its MeSH-indexed subset, meaning that a search relying on MeSH terms only will miss non-indexed but highly relevant content.

The results of the MeSH relevance exercise carried out by eight international experts in integrated care add new insights into the difficulties of searching for integrated care literature. We expected a high proportion of citations retrieved using the MeSH term ‘Delivery of Health Care, Integrated’ would lack relevance to integrated care strategies outside of the United States. This might require the searcher to scan a large number of irrelevant results. Average relevance rating across eight independent reviewers of three sets of randomly selected retrievals, was 42.5%. However, when we added the requirement that citations had to be assessed as relevant by two reviewers, average relevance rating dropped to 24%. Reviewers agreed on a rating of either ‘relevant’ or ‘not relevant’ to integrated care for just under half of the citations, reflecting the highly contextual and personal nature of interpretations of the concept. Taken together, the results of this MeSH screening exercise demonstrate the poor performance of the MeSH term ‘Delivery of Health Care, Integrated’ at retrieving literature deemed relevant by an international group of integrated care experts. This further strengthens the case for developing approaches that facilitate brokered access to the integrated care literature and do not solely rely on the use of the ‘Delivery of Health Care, Integrated’ MeSH term, as it is clear that the current best available MeSH term in PubMed does not facilitate optimal retrieval.

Therefore, we propose the development of an integrated care search filter for PubMed. When objectively derived and validated using a proven methodology, search filters have a known level of performance within the database for which they were designed. The proposed integrated care search filter will use a validated combination of textwords and MeSH terms, thereby ensuring access to both PubMed’s indexed and non-indexed content. Given the significant contribution to the integrated care literature indexed in CINAHL and Embase, we propose that the PubMed search filter be translated for these databases (bearing in mind that the translated search string will not be experimentally validated for those databases).

Our study has a number of limitations. The search strategy underpinning the analysis focused on a small subset of all possible terms associated with integrated care and choices to include or exclude terms were made subjectively. Only highly specific terms associated with the concept of integrated care were used in the search strategy. The reasons for not incorporating a wide range of terms in the search are twofold. Firstly, the aim of the bibliometric analysis is not comprehensive retrieval of all relevant literature (as in a systematic review), rather an inductive approach to identifying the incontestable core of the literature and moving outwards from there to identify other representative terms and concepts. Secondly, integrated care is characterised by a large number of concepts and terms for those concepts. We do not know at this stage of exploration which of these have universal acceptance and which remain contentious.

The bibliometric analysis was also limited to health databases (with the exception of EconLit), and therefore did not include relevant integrated care literature indexed in databases covering related areas such as social care, human resources, finance and information technology.

## Conclusion

Searching for integrated care evidence is difficult. The subject area is conceptually complex and search strategies need to make use of a range of synonyms to ensure adequate coverage. However, increasing the number of terms in a search strategy risks retrieving higher numbers of irrelevant citations. The number of journal citations making reference to integrated care is growing rapidly and these citations are spread across a range of journals. Even using highly precise search terms, searchers can expect to retrieve a proportion of irrelevant citations comprising clinical studies recruiting patients from Integrated Health Services such as Kaiser Permanente. A search of PubMed alone may be adequate if the searcher is only interested in journal articles and willing to forego evidence from the conference literature. We recommend the addition of Embase to a PubMed search if the emerging topics often to be found in conference abstracts are of interest. Embase will also capture additional content from prime titles *Annals of the Academy of Medicine, (Singapore)* and *Value in Health*. CINAHL is useful for accessing the *Journal of Integrated Care* and the *International Journal of Care Coordination*. PsycINFO provides access to additional mental health content but also contains a high proportion of non-journal items.

An integrated care search filter and associated resources will be developed by a project team representing a partnership of the following organisations: the International Foundation for Integrated Care (IFIC) (https://integratedcarefoundation.org/); Central Coast Local Health District (CCLHD), New South Wales, Australia (www.cclhd.health.nsw.gov.au/); The University of Newcastle, NSW (www.newcastle.edu.au); and Flinders Filters, Flinders University, South Australia (http://www.flinders.edu.au/clinical-change/research/flinders-filters/). This resource will be made freely available to researchers, practitioners, and policy makers to improve access to the integrated care knowledge base.
